# The Swedish Psychotrauma Society: joining forces for a national psychotrauma platform from a multidisciplinary and holistic approach

**DOI:** 10.3402/ejpt.v6.28546

**Published:** 2015-06-11

**Authors:** Vania Ranjbar, Monika S. Näslund, Emme-Li Vingare, Christina Hagelthorn, Liselotte Englund, Ingvar Karlsson

**Affiliations:** 1 Angered Hospital, Gothenburg, Sweden; 2 Department of Public Health and Community Medicine, University of Gothenburg, Gothenburg, Sweden; 3 Crisis and Disaster Psychology Unit, Academic Primary Health Care Centre, Stockholm County Council, Stockholm, Sweden; 4 Department of Social Work, Linnaeus University, Växjö, Sweden; 5 Swedish Psychotrauma Society, Sweden; 6 Department of Environmental and Life Sciences, Karlstad University, Karlstad, Sweden; 7 Department of Psychiatry and Neurochemistry, University of Gothenburg, Gothenburg, Sweden

Events such as the discotheque fire in Gothenburg, Sweden, in 1998 and the 2004 tsunami in Thailand, in which many Swedish citizens’ lives were lost, exemplify events of traumatic character with implications for different professions in Sweden (Arnberg, Hultman, Michel, & Lundin, 2013; Wahlström, Michélsen, Schulman, Backheden, & Keskinen-Rosenqvist, 2013). Such events highlight the need for Swedish research on psychotraumatology to meet needs arising in connection with such traumas. Also events in Sweden's immediate vicinity, such as the terrorist attacks in Oslo and in Utøya in 2011, can be considered to have impact on Swedish society and its emergency preparedness. Furthermore, the current globalisation is increasing the need for knowledge on psychotraumatology in Sweden not least because of the increasing immigration to the country from war-torn regions (The Swedish Migration Agency, 2014), which entails a large number of traumatised individuals coming to Sweden. Awareness of posttraumatic reactions as a result not only from war or disasters but also from everyday violence and abuse is closely related (Chrisman & Dougherty, 2014). The lifetime incidence of posttraumatic stress disorder in Sweden is estimated to be 5.6% (Frans, Rimmö, Åberg, & Fredrikson, 2005).

Today, in Swedish universities, there is ongoing research directly related to psychotraumatology as well as research in closely related topics and disciplines. The following are some examples. At Uppsala University, in the Department of Neuroscience and its National Centre for Disaster Psychiatry, as well as in the Department of Psychology, research is being conducted on reactions to traumatic events, such as posttraumatic stress disorder, and factors related to risk, resilience, and recovery from serious incidents (Arnberg, Bergh Johannesson, & Michel, 2013; Arnberg, Michel, & Johannesson, 2014; Lundell et al., 2013). At Karlstad University, research is being conducted on media reporting of disasters and on journalists’ stress reactions when reporting on disasters (Englund, Forsberg, & Saveman, 2014). Research on disaster psychology is also conducted at Karolinska Institutet in collaboration with the Crisis and Disaster Psychology Unit at Academic Primary Care Centre, Stockholm County Council (Wahlström, Michélsen, Schulman, & Backheden, 2013; Wahlström et al., 2013), and at the Prehospital and Disaster Medicine Center in Region Västra Götaland (Khorram-Manesh et al., 2015). Also at the Swedish Defence University and CRISMART—Crisis Management Research and Training—researchers contribute towards advancing the field of crisis management and psychosocial support after trauma (Boin & Bynander, 2015; Enander, Hede, & Lajksjö, 2015; Fischbacher-Smith, Stern, Deverell, Fors, & Newlove-Eriksson, 2014; Nilsson, Sjöberg, Kallenberg, & Larsson, 2011).

Researchers at the Department of Psychology at Stockholm University address the issue of trauma and memory from a forensic psychology perspective, such as how children and adults who have experienced or witnessed traumatic events remember and report such experiences (Azad, Christianson, & Selenius, 2013; Leander, Granhag, & Christianson, 2009). Within the area of child psychiatry, researchers at the Faculty of Health Sciences, Linköping University, conduct research on children and young people who are at risk and/or traumatised in order to identify symptoms through various screening forms for the implementation of rapid and relevant treatment (Kjellgren, Svedin, & Nilsson, 2013; Tingskull et al., 2013). Research focussed on refugees and refugee traumas is carried out at the Red Cross University College and the University of Gothenburg (Ascher & Hjern, 2013, 2014).

As for practitioners, a number of various professions are working with, for example, traumatised refugees, asylum seekers, and undocumented immigrants, or with survivors of sexual assault and domestic violence or serious accidents. They report the need for further education on and enhanced competence in the sequelae of psychological trauma and the management thereof within the healthcare system, as well as the need for knowledge exchange.

Hitherto, however, there has been a lack of a national platform that can contribute to added value through knowledge exchange, continuing professional development, and opportunities for cooperation—not only at annual gatherings but also continuously. There are networks such as ESTD (European Society for Trauma and Dissociation) Sweden and EMDR (Eye Movement Desensitization and Reprocessing) Sweden; however, these societies are restricted for natural reasons, being focussed on a specific type of trauma (complex/chronic trauma and dissociation; ESTD) or a certain type of treatment (EMDR). Thus, there is a need for a national, continuously available platform that unites researchers, students, and practitioners within psychotraumatology, and that considers all aspects of traumatic experiences from a multidisciplinary and holistic approach.

In addition, Sweden is currently one of the few countries in northwestern Europe without a formal connection to the European Society for Traumatic Stress Studies (ESTSS). With the ESTSS’ role as the European coordinating organisation within the subject of psychotraumatology, a Swedish connection to the ESTSS and its affiliated organisations would undeniably facilitate the stimulation and strengthening of psychotraumatology research in Sweden—not least through hosting of future ESTSS conferences in Sweden. A well-established Swedish psychotrauma society would in turn enable such ESTSS affiliation. The Swedish Psychotrauma Society (“Svenska Psykotraumaföreningen”) aims to meet the above-discussed needs and objectives.

The Swedish Psychotrauma Society was formed in 2013. Its objectives are to increase and disseminate knowledge on psychotraumatology by creating a network of professionals and individuals who are active and/or interested in the subject, promote research and training in the field, and collaborate nationally and internationally on issues relating to psychotraumatology. The society is open to researchers, students, practitioners, and others alike with an interest in psychotraumatology, thereby encouraging multidisciplinary and holistic work and collaboration as well as being one of its kind in Sweden within this field. Not least, the society strives to be a link between research and practice.

To this effect, the society organised its first scientific conference in May 2015, with a grant from The Swedish Foundation for Humanities and Social Science. The conference aimed to offer internationally renowned psychotrauma researchers as keynote speakers (Litz, 2015; Newman & Drevo, 2015; Olff, Van Zuiden, & Bakker, 2015) and gather a nationwide audience consisting of professionals and researchers from various disciplines. By establishing a Swedish psychotrauma society and inspire and strengthen the field of psychotraumatology in Sweden, it is hoped that the Swedish Psychotrauma Society during the coming year can become affiliated to the ESTSS and commence collaborations with its European counterparts.

This conference was funded by a grant from The Swedish Foundation for Humanities and Social Sciences (F14-1747:1).
